# Acceptability and feasibility of a brief intervention to enhance resilience among young people and their families in India and Kenya

**DOI:** 10.1017/gmh.2024.87

**Published:** 2024-10-18

**Authors:** Kamaldeep Bhui, Debasish Basu, Sugandha Nagpal, Victoria Mutiso, Renjith Pillai, Kristin Hadfield, Zelna Lauwrens, David Ndetei

**Affiliations:** 1 CHiMES Collaborative, Department of Psychiatry, Nuffield Department of Primary Care Health Sciences, Wadham College, University of Oxford, Oxford, UK; 2 WPA Collaborating Centre, Oxford, UK; 3 Global Policy Institute, QMUL, London, England; 4 Oxford Health and East London NHS Foundation Trusts, London, UK; 5 Department of Psychiatry, Postgraduate Institute of Medical Education and Research, Chandigarh, India; 6 Jindal School of International Affairs, O.P. Jindal Global University, Sonipat, Haryana, India; 7 African Mental Research and Training Foundation, Nairobi, Kenya; 8 Trinity Centre for Global Health, School of Psychology, Trinity College Dublin, Dublin, Ireland; 9 University of Oxford, Oxford, UK; 10 Department of Psychiatry, University of Nairobi, Nairobi, Kenya

**Keywords:** child mental health, community-based initiatives, cross-cultural, global mental health, parental involvement, resilience

## Abstract

Enhancing resilience is one way to prevent future mental illnesses and encourage recovery in the face of adversity. To develop and test the acceptability and feasibility (A&F) of a combined family and individual resilience intervention in two rural/semi-rural low-income settings in India and Kenya. We developed a five-session intervention including Life Skills Education (LSE) and a model of family resiliency. Among adolescents aged 14–16 years and their families in India and Kenya, we collected socio-demographics and audio records of delivery and undertook a process evaluation. Due to COVID-19, we developed a hybrid intervention. The facilitators and participants preferred the in-person model. *India:* Of 17 families, 10 fully completed the intervention. They identified three critical components: 1) story-telling, 2) cooperation and working together and 3) expressing feelings. *Kenya:* All 15 families completed the intervention. Critical elements were 1) seeing social value in learning to make good decisions, 2) promoting an optimistic view of life, 3) hearing stories that resonated with their situation and 4) enhancing family performance through knowledge-building. We mapped the active ingredients, showing fidelity and acceptability. The intervention showed promising A&F parameters. Flexibility and local adaptation were important for delivery.

## Graphical abstract

**Figure:**
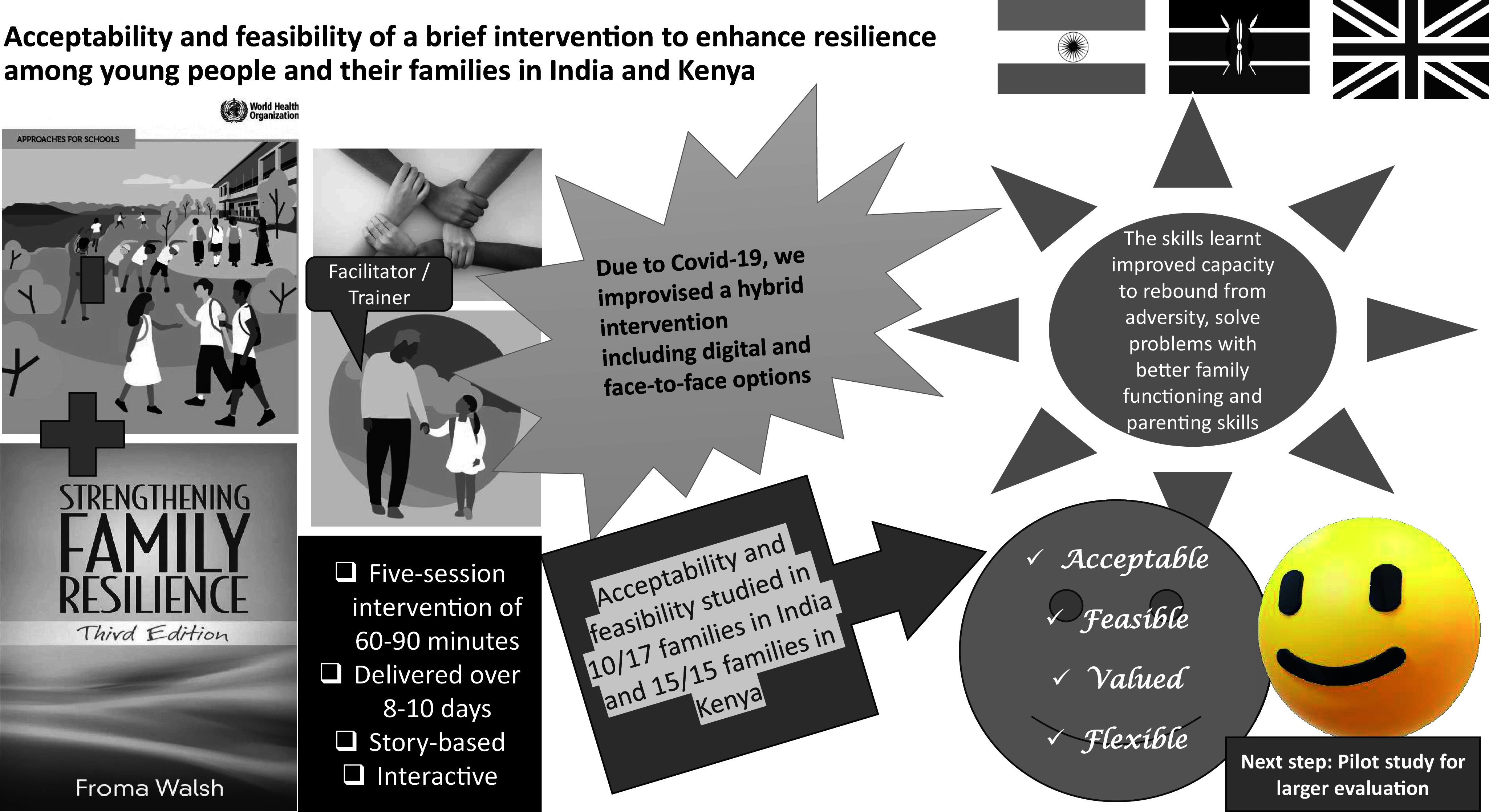

## Impact statement

Mental health problems are common, with 75% of people expressing distress by the age of 24. Early intervention and prevention are therefore essential, not least as in low-resource settings there are insufficient specialist professionals or public services to meet the need. Many young people never meet the diagnostic threshold by which public services provide care. Our study attempted to integrate two ways of supporting young people by combining life skills education with a family approach to enhancing resilience, so supporting young people and their families to problem solve. We successfully designed an integrated intervention showing that markedly different contexts in India and Kenya, and different delivery methods, were as acceptable and feasible and appeared to deliver the relevant processes to help families and children have better conversation and perspective take. The potential of the intervention for scale up requires further testing in different countries working towards a trial; however, the impact of the approach was found for participants and holds important lessons for policy makers seeking to provide support in low-income settings. We produced a manual (https://osf.io/9uxz3/?view_only=cb90ab17234e456dbe7e03c06f7e46a5), which can be accessed free of charge online, for others to use and develop and test. Specifically, in India and Kenya, the levels of poverty, stigma, rurality and isolation, and lack of health literacy are drivers of poor mental health and restrict help seeking or the provision of care, even where it exists. Our preliminary work impacts on participants in the programme and built capacity in our teams and we now have training and delivery methods that can be adopted by others. The project has also skilled local people to deliver the intervention and so created capacity for future work and ongoing knowledge diffusion.

## Introduction

Preventive approaches can be cost-effective and reduce the burden of mental illnesses in the community (Colizzi et al., 2020). This requires a shift of policy and practice from treating established mental disorders to also promoting mental health in the population. This is even more pertinent for low- and middle-income countries (LMICs), where the levels of poor mental health and needs for care far outstrip the available resources for treatment services (Alonso et al., 2018; Sankoh et al., 2018). Over 50% of adult mental illnesses are manifest by the age of 14 and 75% by the age of 24 (Kessler et al., 2005), advocating for prevention and early intervention. Early life experiences and adversities can lead to mental disorders later in life; therefore, preventive interventions aimed at enhancing resilience to adversities are logical and necessary (Patel et al., 2008). Resilience, contrary to what was thought initially, is not an inherent, innate, immutable personality “trait” but rather a dynamic multi-level systemic “process” that is changeable over time. In turn, resilience impacts mental health, adjustment to and thriving in the face of adversity and stress (Ungar and Theron, 2020; Masten et al., 2021). An important corollary of this reframed conceptualisation of resilience is that resilience, both at the individual and family level, is modifiable and can be improved by intervention.

### Life skills education

One approach to enhancing resilience in young people is the World Health Organization’s (WHO) Life Skills Education (LSE) (Ndetei et al., 2019; Sherif et al., 2023). This promotes psychosocial competence, defined as “a person’s ability to deal effectively with the demands and challenges of everyday life. LSE enhances a person’s ability to maintain a state of mental health and to demonstrate this in adaptive and positive behaviour while interacting with others, in culture and environment.” The 10-component LSE module (see Table 1) was designed as “a school-based programme for children and adolescents, to be taught in a supportive learning environment.”

**Table 1.** Ten life skills in WHO model

Self–awareness Empathy Critical thinking Creative thinking Decision–making Problem–solving Effective communication Interpersonal relationship Coping with stress Coping with emotions

While laudable, there are several problems with an exclusive school-based approach. There is intense competition for education and employment, so academic success is often defined as getting through competitive exams and job placement rather than enhancing life skills, social competencies and relationships. Therefore, LSE may be seen as a lesser priority in curricula. There are motivational challenges as well: for example, a lack of student interest due to academic burden and short-term ambitions; the imposition of LSE may be perceived as an additional burden on teachers. There are practical/logistic challenges; for example, time constraints due to academic and teachers’ competing priorities; poor pedagogy; lack of proper training for teachers to grasp the ideas of LSE programmes. Additional challenges include the teachers’ own developmental needs, like assessment skills and social and emotional competence. Furthermore, LSE may become an extracurricular option rather than a core activity. Furthermore, school and family systems are often distinct with variable and sometimes not much interaction between the two. Therefore, home difficulties may be overlooked. If families are not involved, they may not perceive this type of education as relevant, and therefore, they may oppose or undermine its implementation. Furthermore, many children are out of school, even at the primary level, in many countries, because of a lack of availability of school places, if school fees are unaffordable or where poor mental health leads to exclusion because of externalising symptoms (conduct and behavioural problems) or inability to engage due to anxiety and depression. If we focus solely on school-based provision, then we miss some of the most vulnerable young people.

### Family influences

Given the need to expand our focus beyond the school and the importance of family structures in LMICs, we sought to develop a model to be implemented in family settings. Family values, practices and rituals; communication patterns; the quality of interpersonal relationships, bonds and cohesiveness; common family life-events, including financial or health adversities, all impact the child and young members in the family. This may be especially true for those in collectivistic societies, where the family has a strong influence on individuals and any decisions that might affect the family. Families are also a vital resilience resource (Hadfield and Ungar, 2018; Theron, 2020), especially in resource-constrained LMIC contexts. The social protective mechanism of families can be best captured by the concept of family resilience, which is defined as “the ability of the family, as a functional system, to withstand and rebound from adversity” (Walsh, 2011). The approach encompasses how the family can process and manage stressful conditions, reorganise thinking and actions in crisis scenarios and identify the risks and strengths in dealing with adversity. In an LMIC setting, locating the intervention in the family may be more practical than a programme located only in schools since it builds on the social protective function of families and allows young people and families to pool their resources. Of course, there will be limitations, for example, if the carer is not a family member but part of the formal care system for looked-after children, if the carer themselves escaped abusive experiences and neglect and has care needs or if the carer is themselves inflicting harms, for example, exploitation into child labour or maltreatment.

### Aims

In this study, we a) developed a combined family and individual resilience intervention and then tested its acceptability and feasibility (A&F) in India and Kenya, (b) tested if this intervention can be delivered at the community level in families and (c) we wished to optimise the intervention in order for it to be scaled up and evaluated in a larger study in future. Although we had not anticipated the COVID-19 pandemic, clearly this influenced our approach and offers learning about implementation in crises.

## Methods

### Developing and refining the intervention

This study was conceived during a series of three intensive sandpit meetings in Delhi, Mumbai and London in 2019, as part of “Resilient Futures India Initiative” (RFII). RFII is a multilateral partnership initiative between India and the UK, intended to potentially benefit Commonwealth-affiliated LMICs. Our team was based in India, Kenya, Ireland and the UK. The RFII was led by Queen Mary University of London’s Global Policy Institute (who funded the work), with support from the Oxford India Centre (Somerville College) for Sustainable Development, as well as the Commonwealth Secretary General. The intention was to develop low-cost interventions suitable for roll-out across LMICs. Inherently there is tension between identifying a low-cost and low-risk intervention that offers benefit in context of the sheer scale of need in terms of poverty, levels of mental health problems and demand versus taking the intervention or policy through all the stages of development, including an RCT at the end, which would delay implementation and innovation (Victora et al., 2004; Indig et al., 2017; Foti et al., 2020). Either way, early design and adaptation and feasibility and acceptability steps are essential before proceeding to a trial (Wight et al., 2016). These decisions are practical, ethical and political questions, given some interventions (policy, social or organisational) are implemented without any evaluation and evidence is sought afterwards, and at times of great change or when faced with complex scenarios and processes, decisions will be made at a policy level with whatever evidence is available, and quality improvement and design methodologies might be prioritised over trials (Crowe et al., 2022).

In the context of these dilemmas and challenges, the initial meetings (in Delhi, Mumbai and London) explored more general and formative questions, such as the main study idea, focus, locus and modus. These were mostly in-person meetings (the London meeting was partly hybrid). Later meetings, more focused on the intervention and its delivery modes, etc., were mostly online meetings, given the COVID-19 pandemic. There were 5-6 such meetings, 3-4 among the investigators in India and Kenya separately and 2-3 meetings with the whole group online. The manual was completed after local early field testing and conversations with local potential participants and partners. Later refinements were made over Zoom meetings, as the lockdown meant we were not able to meet in person. We considered optimal formats for delivery, recruitment and retention strategies, and which outcome measures might be suitable. At every step, we attempted to keep the local context in mind in terms of rurality/urbanity, socioeconomic status, poverty, literacy level and cultural influences.

While developing the approach, we followed the important guidelines issued by the UK Medical Research Council (MRC) on developing and evaluating complex interventions (Craig et al., 2008). We were especially guided by the recent elaboration of the development phase of such interventions, published just around the time we were developing our intervention (O’Cathain et al., 2019). Thus, we undertook a *review of the published* literature (Basu et al., 2020) to inform the development of the intervention and *evolve a theory of change* (programme theory). The review revealed much evidence on enhancing resilience and mental health of children and adolescents by integrated school‑ and family‑based approaches, with a focus on LMICs. Based on the limited literature available from LMICs, the review concluded that “such interventions are at least partially effective, and potentially feasible, despite challenges.” The review endorsed the need for the following:

- multi-component interventions.
- involving familial and child resilience.
- engaging trained lay counsellors and peers rather than depending solely on teachers and health practitioners.
- working within the needs and context of local resources and cultural influences. We explored the feasibility of technology-based interventions given the COVID-19 pandemic coincided with the research (Basu et al., 2020).

In our team discussions, we iterated a unified programme theory using concepts proposed by Walsh (Walsh, 2003, 2011; Froma Walsh, 2016; F Walsh, 2016) on family resilience domains (*belief systems, organisational processes and communication processes*) and their nine components (*meaning-making, hope, spirituality, flexibility, connectedness, resources, clear information, emotional sharing and collaborative problem-solving*). These provided the bedrock for developing the family resilience components of the intervention. We combined this with the ten components of LSE (see Table 1), and components of psychological resilience found in the literature (World Health Organization, Division of Mental, 1994). A large number of existing training and resilience-fostering programmes and their manuals were consulted (see Table 2).

**Table 2.** Training and resilience resources. A large number of existing training and resilience-fostering programmes and their manuals were consulted during the development of our intervention package. These included the following:

A positive psychology programme (Ndetei et al., 2019; Ungar and Theron, 2020) A Family Coaching programme (developed by Zelna Lauwrens) (Lauwrens, 2010) Life Skills Education programmes developed in India, Kenya and elsewhere (World Health Organization, Division of Mental, 1994) FOCUS programme for military and veteran families in the USA by William Saltzman (Saltzman, 2016) Wellbeing and Resiliency Program in Alberta, Canada (Alberta, 2019) Strengthening Families Program by Karol Kumpfer in the USA (Kumpfer and Brown, 2019) Resilience and Coping Intervention by Sandra Allen (Allen et al., 2016) Resilience Research Centre in Canada and Family Competence Program in Spain A large resource–rich compilation of 23 evidence–based family skills training programmes published by the United Nations Office on Drugs and Crime (UNODC, 2009). It was noted, however, that the published evidence mostly came from the high–income countries and the Western world, with very different sociocultural, family and economic contexts to those in India and Kenya.

**Table 3.** Socio-demographics of parents

**India**		N	%
Gender of participants	Male	0	–
	Female	8	100%
Level of education	Primary	3	37.5%
	Secondary	3	37.5%
	Tertiary (college)	2	25%
Age	<20	–	–
	20–40	7	87.5%
	>40	1	12.50%
Occupation	Business person	1	12.50%
	Casual labourer	–	–
	Community health worker	1	12.50%
	Farmer	–	–
	High school student	–	–
	House help	–	–
	Housewife	6	75%
	Mason	–	–
	Retired teacher	–	–
**Kenya**	–	N	%
Gender of participants	Male	8	36.4
	Female	14	63.6
Level of education	Primary	17	77.3
	Secondary	4	18.2
	Tertiary (college)	1	4.5
Age	<20	0	–
	20–40	3	13.6
	>40	19	86.4
Occupation	Business person	3	13.6
	Casual labourer	3	13.6
	Community health worker	2	9.1
	Farmer	5	22.7
	House help	1	4.5
	Housewife	4	18.2
	Mason	3	13.6
	Retired teacher	1	4.5

Primary (1–8), secondary (form 1–4) and tertiary (college).

**Table 4.** Socio-demographics of adolescents

**India**		N	%
Sex	Male	4	40%
	Female	6	60%
Education	Primary	4	40%
	Secondary	6	60%
Age	Age (13–16), years	10	100%
**Kenya**		Count	Column N %
Sex	Male	6	40%
	Female	9	60%
Education	Secondary	15	100%
Age	Age (15–18), years	15	100%

Primary (1–8 grade) and secondary (9–12 grade)

**Table 5.** Venues, samples, methods and findings from India and Kenya

		India	Kenya	Explanatory notes
Setting	Locality	Semi–rural	Rural	
	Families	Community living; many migrant families	Community living; mostly stable families?	
	Setting of intervention delivery	Family residence	Community meeting place?	Initially planned for a combined school plus community setting; but the pandemic–caused school closure forced us to change track and deliver it solely in the community, at the residence of individual families (India) or community place (Kenya)
Intervention: material	Intervention package	Initially 10 sessions but later trimmed down to five sessions	Initially 10 sessions but later trimmed down to five sessions (same package used as in India)	The package was derived by combining the essential aspects of Who Life Skills Education (for the children) and Walsh’s Family Resilience Framework (for the families). Each session contained some parts of both these elements
	Translated to local language?	Yes (Hindi)	Yes (Swahili)	
Intervention: delivery	Delivered by	Research associates (RAs)	Community health workers (CHWs)	Both trained with audio recording and feedback
Intervention: recruitment	By whom?	RA contacts and networking	CHW contacts and networking	CHWs know their communities better, and there is higher access and trust; hence recruitment, working and completion rates are better. Issues may be constraints on their time and resources (already overworked)
Outcome: feasibility	How many recruited?	17	15	See comments above; perhaps the better in–person access, trust and practical logistics utilised by the CHWs in Kenya explain a better completion rate. Further, as explained in the text, the major dropout in the Indian setting was from migrant families who left or had difficulties during the pandemic, and because many of these sessions had to be conducted online, with its own challenges
	How many completed all five sessions?	10	15	
Outcome: acceptability	Perceived usefulness	Yes	Yes	When used on a 1–5 Likert scale (from 1 as “not at all” or “strongly disagree” to 5 as “almost always” or “strongly agree”), the median scores for these items were 3.5 or 4
	Perceived application (translatability) to real–life situations	Yes	Yes	
	Would they advise/refer others to take up this intervention?	Yes	Yes	When used on a 1–5 Likert scale, the median scores for these items were 3 (would advise/refer 3–4 families known to them)
Feedback	Format	Participants: interviews with participants (all 10 completed families), and also semi–structured questionnaire as pre–post assessment (for four completed families)	Participants: focused group discussion for all 15 families, and separately for CHWs

**Table 6.** Mechanisms and programme theory

Programme theory: LSE, Walsh’s family resilience and Framers’ mechanistic learning		
LSE components	*Walsh’s family resilience*	Our five-session intervention: evidence from evaluation
Self–awareness Empathy Critical thinking Creative thinking Decision–making Problem–solving Effective communication Interpersonal relationship Coping with stress Coping with emotions	Three key elements: Belief systems Organisational processes Communication processes Nine components: Meaning–making Hope Spirituality Flexibility Connectedness Resources Clear information Emotional sharing Collaborative problem–solving	Empathy and perspective taking Understanding each other’s burden and roles Optimism and awareness that tomorrow will be here and there will be a solution Sharing is good and asking for help, and parents will help Knowledge about family function – how to interact in a family Ability to identify what decisions–behaviours were not helpful Kindness in how to respond to a child not getting out of bed, or a single parent who is overwhelmed Avoid quarrelling and keep the peace Asking questions and finding out what is wrong Not to worry (about school fees) and focus on exams Skills help bounce back Confidence in how to run family life Unity in family is important Cooperation Good explanation from research team Storytelling Aware decisions may not have been ideal, and forgive oneself and try to improve them

We identified gaps in existing programmes, and a need for culturally based interventions; thus we worked through several iterations developing an intervention that was brief, simple to apply and appropriate to the local cultures and contexts in Kenya and India. We incorporated interactive elements and focused specifically on enhancing resilience to adversities at the child and family levels.

### The intervention

The initial intervention proposed a participatory learning process in 10 sessions, over 2–3 months, with a possibility of booster/follow-up sessions. We undertook further consultations with local communities, who recommended a shorter version (hence we produced a five-session version). We were also asked to adapt the approach to the COVID-19 pandemic as access to communities varied from week to week as did health protection advice in each country. We took account of feedback from colleagues in the Global Policy Institute as well as local stakeholders, as we finalised the protocol.

The finalised intervention consisted of essential elements of both LSE and Walsh’s family resilience approach. The intervention included in-person and/or telephonic/video option (McEwen et al., 2020) and storytelling; for example, where a scenario of a lower-middle-class working family suddenly facing the COVID-19 crisis was used to guide the discussion on resilience. This was combined with elements of the LSE geared towards the children in the family. The five sessions of 60–90 minutes duration addressed the following anticipated mechanisms that were shared by child and parent: *rapport building setting the agenda* (first session); *belief systems, organisational processes and communication processes* (second through fourth sessions); and *closure with feedback* (last session).

There were unexpected and unpredictable lockdown periods of differing duration and forced compliance. We had to adapt, be agile and accept that a fixed and predetermined schedule developed away from the sites was not likely to work. This principle of adapting to local community assets and preferences means an intervention can be applied flexibly where required. We ended up not restricting the local site leads from following the sentiments of their local communities, notably influential local community figures and leaders, while taking feedback when setting up the study. Although this might mean different approaches were taken in each site, we felt this was a strength, making it more likely that interventions would ultimately be adopted at the time of implementation, rather than enforcing an approach that local communities did not favour and would not take up.

### Facilitators

In India, we trained two local residents who had completed or were pursuing their postgraduate education in psychology and counselling. In Kenya, we recruited and trained five community health workers who were local residents. Thus, we adhered to our overall principle by engaging local lay providers and professionals, allowing us to build capacity and develop a sustainable approach. A manual was developed to support the training of facilitators. The training included didactic talks, role-playing and feedback. The facilitators were key to not only delivering the intervention but also establishing and maintaining connections with the families throughout the project. In both sites, the facilitators were trained (three days in India and a week in India), and skills were strengthened through role-play and feedback. The research team provided feedback on the role-plays and delivery skills and were satisfied the facilitators were delivering the designed intervention. The facilitators translated all materials.

### Testing A&F

We planned to optimise the intervention, test acceptability and feasibility. The acceptability and feasibility parameters were drawn from the existing literature and guidelines in this area (see Annex). (Bird et al., 2014; Skivington et al., 2021) This acceptability-feasibility framework was used flexibly, gathering the information from process interviews or by questionnaire, as preferred by the researchers and participants. The facilitators audio recorded sessions which were used for quality and fidelity checks by the research team, and feedback was provided. At the end of five sessions, the family views on acceptability and feasibility were sought and recorded in accord with the parameter set out in Annex.

**Figure 1.**
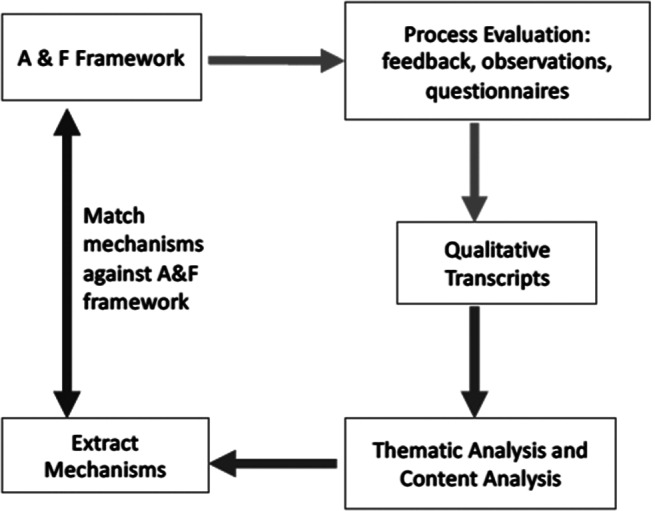
Approach to testing A&F.

We used the A&F process evaluation to understand how participants used the intervention and what recommendations they had, but importantly, we also wanted to determine their views about how the intervention was helpful (mechanisms) or not in specific contexts (Moore et al., 2015; Limbani et al., 2019). In so doing, we drew on traditions of thematic analysis of qualitative data in process evaluations (Moore et al., 2015; Limbani et al., 2019; Perry et al., 2024), although quantitative and qualitative data from multiple sources can be used (Bowden et al., 2020). Thus, we originally undertook a thematic analysis and identified narratives that reflected social, psychological and intervention-related mechanisms and specific verbatim content (Figure 1).

Although we did not undertake a more formal critical realist analysis, this approach endorses narratives as reflecting real-world data rather than subjective experiences to be dismissed, and that complex interventions may trigger specific mechanisms of effect that vary by context (Hua et al., 2023; Mukumbang et al., 2023). The outcomes thus may vary, and this is not seen as problematic but rather more informative about how an intervention works in the real world and which intermediary mechanisms are responsible. The approach does not seek to identify universal truths, or a fixed inflexible intervention; rather it accommodates complexity of intervention take-up and anticipates the programme theory will be refined in future work. Using these principles, the mechanisms were identified in the transcripts (highlighted by underlining relevant sections of the narrative thematic analysis, alongside verbatim quotes to illustrate certain points). We included a pre-post questionnaire (PHQ2 and GAD2) (Kroenke et al., 2003; Hughes et al., 2018) in one site (India) to assess feasibility of data collection rather than analyse the results for determination of effectiveness or efficacy.

### Research settings

India: The study was conducted in two villages, Khewra and Indra colony, Sonipat district in Haryana. Sonipat is located 20 km from India’s capital city, Delhi. It is known as an education city since it houses universities such as Ashoka University, Rishihood University and O.P. Jindal Global University. In the last two decades, the economy of Sonipat has transformed from being primarily agricultural to more industrial. At the time of the study, Sonipat had a population of 278,149, of whom 176,346 were migrants from elsewhere in India. In Khewra village, most of the population is native to the village and agriculture is the primary occupation. Indra colony is locally referred to as the migrant colony since it mostly houses migrants from Uttar Pradesh (UP), Bihar and Punjab.

Kenya: The study took place in Makueni County, which is one of the 47 counties in Kenya. Makueni County covers an area of 8,009 km² and is located 106 kilometres away from Nairobi. The main economic activity carried out in this region is agriculture. It has a population of 987,653. In total, 58.4% of residents are between the ages of 15 and 64. This study site had participated in the team’s previous research on resilience among children in Kenya (Ndetei et al., 2019).

### Participants

Adolescents were aged between 14 and 19 years. The aim was to complete all five family sessions in 8–10 days. At the end of the sessions, the research assistants asked for sociodemographic information and the families were given a small token of appreciation.

### Study sites and execution (see Table 5)

In India, two facilitators, one male and one female, were recruited to deliver the intervention. They received a three-day online training to orient them to the research project, research methods and ethical issues of fieldwork. Thereafter, they engaged in an iterative process to translate the manual from English to Hindi and align it to the literacy level and dialect of the population. They also conducted a series of role-play exercises to refine the delivery of the intervention, under the supervision of senior researchers. It was delivered in community settings with young people and at least one adult family member. While we started delivering the intervention at the homes of families, due to restrictions imposed by the pandemic, the intervention began to be delivered at the house of one of the facilitators who lived in Khewra village; the other facilitator attended remotely through an audio call. The physical presence of a facilitator from the area enhanced the understanding of families. The facilitators reported that online sessions would have been difficult for the families to understand, engage and trust. In Kenya, local consultation was followed and different approaches were tested. Translation was done in the local language (*Kamba*). The final version was achieved through forward and backward translation with meetings with translators to iron out disparities. The translated intervention was then shared with the facilitators to determine flow, acceptability of the language and relevance before final commitment to the translated intervention. The research team trained five local lay providers (community health workers) over one week, in their local language and through the use of role-plays. In the delivery phase, they received weekly supervision by the research team to resolve any challenges.

The families were divided among our trained lay intervention providers, so each provider had three families to work with. Sessions were conducted in the evenings after the students were from school. This was done face-to-face within the compound of the participants while observing COVID-19 regulations.

### Ethics

Ethical approvals were secured from O.P. Jindal Global University in India (PGI/IEC/2020/000255) and the Maseno University Ethics Review Committee in Kenya (MSU/DRPI/MUERC/00927/21).

## Results

The demographics of participants are presented in Tables 3 and 4.

### India

#### Feasibility

Of 17 recruited families, 10 (58%) completed all 5 sessions. In Indra colony, there were several reasons for variation in engagement and session completion due to the pandemic and other local pressures. Initially, while there were 8 families recruited from Indra colony through community contact via a local non-governmental organisation (NGO), during the second wave of the pandemic in March 2020 a number of these migrant families went back to their home villages and thereafter were difficult to contact. Out of these 8 families, 4 families withdrew completely, 3 completed one in-person session and 1 family completed all sessions (some of these sessions were in person). Khewra village: The facilitator used peer and community networks to recruit nine families in May 2020. These nine families completed all five sessions. In Indra colony, pre-post assessments were completed by four families (23%). The 10 fully engaged families answered demographic questions and provided feedback.

The average monthly income of the 10 families was slightly above the national monthly income. In addition, six of the families owned agricultural land and exhibited diversified livelihoods. In these families, in addition to the adult men working in Sonipat’s industrial sector as labourers, drivers, guards or lab technicians they also had agricultural income. Most women were housewives. Overall, the respondents demonstrated higher levels of economic security than the families who dropped out. In addition to the challenges introduced by the pandemic, a few adult men in the dropout families were juggling multiple jobs and long working hours.

During the second wave of the pandemic, most of the migrant families in the study returned to their villages in other Indian states. Given the high fatality and infection rates, and low vaccination rates, for safety reasons, the facilitators were not permitted to visit families in Indra colony. Many of these families did not take calls or adhere to appointments. Despite much effort to retain the families recruited in Indra colony, we had little success.

At the onset of the COVID-19 pandemic, lockdowns were strictly enforced in India. We developed a hybrid *digital face-to-face* intervention. The transition to the hybrid model also entailed challenges; for example, there was unstable internet connectivity. One facilitator could join only through a WhatsApp audio call, and still he could not hear everything being discussed. The Khewra facilitator had to communicate between families and the remote facilitator. Connectivity challenges created a disjuncture in the flow of the sessions. The remote facilitator could not observe the body language, gestures of family members and the setting of the sessions. This limited the reflections and observations that could be made.

Valuing the in-person model: The facilitators and participants alike expressed a preference for the in-person model. The facilitators felt that if they were both physically present, it was easier to give information and get clearer confirmation of participation from families. In addition, the in-person model enabled greater family participation and engagement from inception. This was hypothesised to be explained by socially and culturally based obligations that motivated family members to attend and respond to the facilitators as they were guests, physically present and had taken time to spend with families. Family members said they had more trust and understanding and willingness to participate given the human contact and the social rituals of drinking and eating together while talking. For example, in explaining this, an adult man (family 7, session 1) said:

*….when someone comes then we should talk to them‥ and like this we think that how in a more better way we should tell this to them…move forward to eat and drink‥ have to look after all these things take care of all this.*

In this excerpt, the participant explained the need to be hospitable and responsive when people visit them. In visiting the homes of families, it was also possible for participants to carry on with their daily chores while conversing with the facilitators. Thus, it required less disruption and effort for the family.

To explain her preference for face-to-face sessions, a child (family 14) said :

*…because we get to understand things much better and there is problem of internet in online sessions.*

However, despite a preference for the in-person model, within a close-knit and small community there were other barriers. In one session (session 1 with family 4), the female facilitator from Khewra was unavailable. When the male facilitator visited, the husband was not at home; the wife and mother were hesitant to meet. The wife considered it inappropriate to speak with an unknown male and was concerned the neighbours might perceive this as breaking conventions around male–female interactions, and they may have become the subject of negative comments. She also had young daughters and wanted to protect them. This meant it was difficult for the male facilitator to continue the session. The session was concluded prematurely with the promise to return another day with the female facilitator.

In addition, an open-door policy meant people freely walked in and out of each other’s houses. In some sessions, the large gathering of people was disruptive, and it was difficult for the facilitators to conduct sessions and assure confidentiality. Furthermore, the houses in Indra colony where migrants lived were congested and in close proximity to the main road. Often migrants were living with their entire family in a single room, and there was a small common space in the middle of the house, where the sessions were conducted. This space was also used by other residents doing household chores. There were background noises, including dishes clattering, bikes, scooters and autorickshaws, making it difficult to hear, record and conduct the sessions smoothly. In conclusion, physical visits to the community and homes of families entailed actively managing and working with the community’s physical infrastructure, and existing cultural and social norms. Yet, digital or telephone contact was fragile and did not engender as much engagement.

#### Acceptability

Positive Feedback: The 10 families with whom all five sessions were completed said they had learned much about family life, how it should work and function and how in a number of ways they can enhance resilience. During the session, participants displayed attentiveness, engagement and retention of information; they were able to recap the learning as the sessions proceeded. For example (session 3, the young male child, in family 13):

*Sir, we have discussed how S’s family had handled the situation, who all have helped them. ……we have read about good behaviour and how we can talk nicely with people ……and how can we control our anger, and … develop a good nature…*

In addition to retaining and summarising the sessions as they were taking place, participants also relayed optimism and hope in their feedback about the sessions overall. For example, an adult (family 12, session 5) said:

*Sessions were very good, we learned that to see, I might have also done some mistakes but I have learned so many things, and most of all the children have learned something good….they must have learned something good that what should be done, how to communicate, to not fight in home, everyone should stay together and talk nicely, it is not that there is no solution for anything, if we can work towards it we can definitely find a solution.*

In the feedback sessions, the participants were able to summarise the main aspects of the sessions and identify their value for their own lives. They proposed the following key elements that helped.

Story telling was well received and helpful; for example, a male child (Family 13) said:

*I like the sessions so much because I get to hear a very good story, and we have learned so many good things that how we should do that, how can we solve the problem, how we can keep our emotions and feelings in control, maintain discipline, and how to empathise with others.*

…*like you explained well, explained with the help of examples. Some things I didn’t know that I got to learn. You have told many things for the children this can be useful for all of us in life.*

The value of the story was also endorsed by the facilitators, as the story allowed them to communicate abstract ideas of family resilience in a relatable format to the families.

Cooperation and working together seemed important; for example, an adult (family 12) said:

*All good! 1^st^ day’s session was good but 2^nd^ day’s session was better. You have told everyone through the story that how to work well. Told about all the people of the house, how to face problems. With the help of story, how can the men, women and children of the house all come together in trouble to overcome the problem.*

Expressing feelings: During the sessions, participants felt comfortable expressing their feelings to the facilitators. These included experiences of mutual care and support but also negative experiences. For example, a young male child (session 1, family 13) said:

*Sir, I have to take care of my mother and Sir if something happens in the house I have to take care of house too and Sir like my mother takes care of me, sometimes she also gets sick so I should also take care of her, that’s all I want to say Sir. I…I Love my Mother!*

### Kenya

#### Feasibility

Recruitment methods: The recruitment approach was influenced by feedback from communities and early conversations with local community leaders. The suggested but structured and unstructured sharing of information with communities well before the study began. This is in contrast to India where recruitment was based on the networks of the co-investigators and research assistants. This approach in Kenya was endorsed when we received feedback from participants. One young participant noted that:

‥*saw something about it through a named community health worker and then later some people came to tell us about this organization.*

Many recipients learned about the programme through word of mouth, and this positively influenced people’s decision to participate. A hybridised community entry strategy comprised structured and non-structured procedures to eradicate misunderstandings also and reduce stigma:

*I asked them what mental health was because I thought it was about mental illness, but they told me that was about me being in a position to make good decisions and persevere when I am experiencing challenges. I was satisfied and happy with the explanation they gave me.*

Some adolescents learned about the programme through their mentors, who were community gatekeepers. The following example shows how the informal spread of information from multiple sources shaped participants’ expectations long before they were admitted into the programme.

Facilitator: *You have just told me what you learned from the program; I want to know how you came to know about the program. Did you hear about it somewhere? Did someone tell you about it?*

Respondent 1: *I know if it is rolled out in so many places it would help a lot of people.*

The attractiveness of the intervention already discussed in community venues could have influenced decisions to attend sessions; all individuals attended all five sessions.

Participants even requested a scaled-up intervention that would allow more members of the family to participate. The unanimous view could be interpreted to mean that the intervention created an immersive learning experience that was relatable to the unmet needs of both the parents and their children as implied by the excerpts below. The facilitators also reported that all families were committed to the training programme and were even asking for a revamped intervention that had more sessions (they sought 10 sessions). The training method was instructor led, where a community health worker delivered a family-centred micro-learning in a household set-up.

#### Acceptability

The in-person training was appropriate and smoothly implemented for the sociocultural context, with participants expressing satisfaction.

*Before I started this program, I would feel like less of a person whenever I had a problem. After I met this organization, I came to realize that* *I should not let a problem put me down because there is always tomorrow, and I will always figure out what to do.*

*When the people from this organization came to our home, I was at liberty to ask questions and share my problems with them. Whenever I had a problem, I would discuss it with one of the teachers, or I would talk to my parents about it and they would help me solve it and I would always feel better after that.*

*Through the program, I learned how to identify my problems, like if I fail my exams I would identify where I went wrong. I also learned how to solve my challenges and help improve my insomnia issues.*

Participants suggested the approach as well as the content allowed them to learn about the malfunctions of their family. This was reflected in the comments where one mother reported:

*I never used to distinguish between right and wrong. My husband would talk to me and I respond badly.*

Another stated: *myself and the child sat down and learned with the trainer. After the day’s training, I learned that our families have got so many challenges and these challenges can be addressed. I also learned that lack of unity can divide a family but when a family functions as a unit, it progresses.*

Participants provided positive feedback about the intervention. They asserted the social value of the programme. For instance, one participant reported that the intervention:

…has helped us change our behaviors. It has taught us to make good decisions and think critically to solve our problems when we get into trouble.

An adolescent also affirmed an optimistic view of life after the training (see excerpt below), a positive outlook that is important in promoting family resilience.

*My life has really changed since this program began. I learned that if you are from a single-parent family, you should respect your parent. Whenever they give you a duty you have to do it, you should not talk back at her, you should always live in peace. If you only have your mother, you should respect the fact that she is playing the role of a mother and a father and that you should always respect he.*

The programme resonated with participants’ situation and context.

*I never imagined training program would be as helpful and important as it has turned out to be. I now know how to live a better life in my family.*

The programme seemed to usefully enhance the performance of the family. All participants attended the five sessions. One respondent noted:

*‥the trainer trained for five days. I understood what he trained on as the information would help build my family. It would also help me run my family matters better and solve problems…Where a child is going through challenges, I can be able to bring him / her closer to myself without anger so that we can solve the problem and if the child was on the wrong can be corrected.*

The intervention was also lauded as a knowledge-building activity that led to a better understanding of how to interact at the family level. Several respondents noted that the programme imparted skills that improved their capacity to rebound from adversity, reflecting the training was strengths based so the participants realised their full potential. This supposition was best reflected among adolescents, where many of them revealed that they learned how to focus on living their life to their full potential without concentrating on socioeconomic disadvantages.

The excerpt below from an adolescent captures the positivity, where they noted that the training allowed them to cultivate a focused, motivated and happy life even amidst challenging conditions. The intervention:

*…has changed my life in terms of my studies. Through the program, I learned that I don’t have to worry about where my school fees or food will come from when I am at school. I learned to ignore all those challenges and concentrate on my studies when I am at school, and that I should always keep a positive mind about everything. Before the program, I would really get worried about how my future will be and the food challenges we have at home and they would only make me fail my exams.*

A better understanding of how to approach parenting was also reported among mothers, where one stated:

*…. I learnt some good lessons. I learnt* *when a child wakes up and appears not to want to interact with others, I should keenly monitor him / her. I shouldn’t rush to quarrel him / her. Once whatever was disturbing him / her is over, I will approach him and find out what the matter was.*

## Discussion

We tested the feasibility and acceptability of an integrated family resilience and life skills module among low-income families living in rural or semi-urban settings in India and Kenya. Overall, the results were encouraging. We identified several key areas of uncertainty and challenge and provided ideas to improve upon the intervention in its next logical pilot phase of testing, followed by full-scale randomised controlled evaluation.

The five-session intervention seemed to be acceptable and feasible to deliver. However, a key lesson was to adapt to local contexts, to each country and to emerging challenges like the COVID-19 pandemic. Delivery required significant flexibility from the local teams. Face-to-face delivery seemed to be most valued, and there appeared to be important mechanisms of effect consistent with the proposed elements outlined in the origin LSE and Walsh models of resilience (mechanistic findings are underlined and summarised in Table 6).

### Mechanistic elements (Table 6)

The important elements include learning together, reflecting on problems and appreciating each other’s perspectives, and parents and children showing more cognitive and emotional flexibility and changing their responses. Hope and optimism in the face of adversity seemed important. Previous studies suggest family–child communication and problem-solving confer benefits on young people, including processes of emotional regulation, cohesion, meaning-making and culturally congruent actions (Ungar and Theron, 2020). Systems approaches to resilience also emphasise community mobilisation, person-to-person support, solidarity groups and dialogue (Arega, 2023). The delivery teams were able to engage families in meaningful dialogue, and people were welcoming and open to consider a new way of thinking about life’s problems. As the facilitators were from the local areas, they were aware of how the intervention might be optimised for cultural and linguistic competency, and they were trusted as local residents. This may have added an element of community activation and trust. In Kenya in particular, faith communities were active places to share information about the project and they helped raise awareness and recruitment. Emphasising multi-systems approaches, it is possible to see our approach as active at a specific level, e.g. individual and family, with additional impacts on social or environment influences (Twum-Antwi et al., 2020), especially during COVID-19 pandemic, when isolation was a significant challenge and community supports were diminished. We were also mindful of considering the additional distress on families during COVID-19 and that task shifting can be burdensome at times of crisis and conflict (Hinton et al., 2019). Thus the preliminary work consulting families and young people and local communities enabled us to support people in the study. We propose more work should be undertaken on how family-focused interventions work for children and their carers, and the impact on schools and wider society, as opposed to school or social interventions (Ungar and Theron, 2020).

The major takeaway points for this feasibility exercise were the following:

- In-person intervention is more feasible and acceptable than online intervention. However, remote intervention (through telephone, mobile and social media apps such as WhatsApp video calls, etc.) is also possible and reasonably acceptable when in-person contact is difficult.
- Implementation of the intervention programme through the local resources available in the community is feasible with the support from personnel with experience in mental health care.
- Involvement of local influential key persons – village leaders, people’s representatives, voluntary agencies, etc. – is likely to facilitate easy access and programme implementation.
- Sensitivity to local culture and customs is important, as is proficiency in local language for better communication and acceptance by the families and community.
- Neutral spaces (school and community halls) or the residence of the participants are preferred venues, though this would need further testing.

### Adaptation and limitations

The programme would have run better had we not to contend with the COVID-19 scenario: COVID-19-related restrictions in movement, during the first and second waves. Lockdowns and their obvious sequelae compelled us to adapt our methodology. While initially, the sessions were being conducted in person, after the pandemic we adopted both online and hybrid modes of delivery. We also suffered the dropout of migrant families who experienced enhanced economic vulnerability during the pandemic and departed for their home villages. Given the unavailability of the initially inducted migrant families, we moved our field location from the migrant colony to another semi-urban locality in the same area. Since one of the facilitators lives in this locality, it was easier to locate research participants. However, this locality houses a largely local population, and due to the difficulty of contacting new research participants, especially migrants, our final sample of participants is more homogenous, included mostly women as carers and may have included a less poor sample, although overall levels of poverty and rurality were high. In addition, the pandemic reduced the possibility of further rounds of recruiting of eligible families.

The feedback on which A&F was assessed included carers and adolescents together. It is possible had they been asked separately that their responses might have differed. However, given the intervention was delivered as a family unit, we felt the evaluation itself should reflect the unit of intervention. We did not find parents and young people saying exactly the same nor differing markedly; rather the feedback itself seemed to reflect learning and ongoing sharing of perspectives – a process begun during the delivery of the intervention.

Only four of ten families completed outcome measures, which we believe is likely to have been influenced by the pandemic. Clearly future roll-outs will need additional work around which outcomes to select and whether they are appropriate and can be completed. Future replication and pilot studies are required before a randomised trial.

Our preliminary programme theory can be tested and refined over time, and we anticipate the intervention itself (including the manual) will need preliminary testing and adaptation in new populations. The challenges opened opportunities for us to adapt in the face of real and tremendous adversities – a true lesson in resilience building for ***us***! Specifically, despite the constraints and difficulties, this exercise demonstrated the following:

- Acceptance of the module by the intervened family members – adults and children as reflected in their feedback.
- Family members were able to relate their real-life experiences to the ideas and story discussed in the module.
- Appropriateness of the programme reported by the research assistants who implemented the intervention.
- We identified mechanisms of value, most importantly problem-solving and learning together, perspective taking, emotional regulation, optimism and learning, even in a crisis such as the pandemic.

## Conclusions

The combined individual and family resilience intervention was well received, and most aspects of feasibility were demonstrated, not least we believe due to extensive engagement with communities before the work began, and also local adaptation and flexibility. We are planning further work development of the intervention, including replication and pilot studies, to inform a future multi-country trial.

## Appendix

**ANNEX:** A&F Prompts

A&F	**Acceptability questions:** learn, follow, apply, helpful things and things to change.	**Feasibility questions:** engagement, number of families responding and completing intervention, feedback on information and consent, barriers, how long did it take and feasibility challenges
**Acceptability**	What did you learn from the intervention about family resilience? What do you think family resilience is? Was the intervention easy to follow? Expand. How will you apply what you have learned from the intervention? If there is a conflict in the family, how will you address it? How they were affected by the intervention The application of the intervention to their life What was the most useful session in the intervention? Why was this useful? What was the least useful session in the intervention? Why was it the least useful? If you could change two things about the intervention, what would it be? Whether they would recommend it to other families.	What were the different ways thought of/tried to contact families? Which one was found to be the most feasible? How many families were contacted in total, what timeline? How many responded? How many underwent how many sessions? How engaged were the recipients of the intervention, and how good was adherence to the five sessions? What did participants think about the information sheet and consent processes? What were the barriers to participation? On an average, how long did it take for participants to complete the intervention? What led to differences in retention (how can we improve the content)? What were the feasibility challenges faced during the second wave of the pandemic? How were these challenges met, and which ones were more successful/feasible and which ones were not?
