# Supplementary material for: Acceptability and feasibility of a brief intervention to enhance resilience among young people and their families in India and Kenya
Source: Glob Ment Health (Camb). 2024 Oct 18;11:e86. doi: 10.1017/gmh.2024.87 (PMC11504944; doi:10.1017/gmh.2024.87)
Supplement: Peer review history

# Editor report: Recommendation: Acceptability and feasibility of a brief intervention to enhance resilience among young people and their families in India and Kenya — R0/PR2

**DOI:** 10.1017/gmh.2024.87.pr2

**Affiliations:** School of Public Health and Preventive Medicine, Monash University Faculty of Medicine Nursing and Health Sciences, Australia

No accompanying comment.

# Editor report: Decision: Acceptability and feasibility of a brief intervention to enhance resilience among young people and their families in India and Kenya — R0/PR3

**DOI:** 10.1017/gmh.2024.87.pr3

**Affiliations:** London School of Hygiene & Tropical Medicine, United Kingdom of Great Britain and Northern Ireland

No accompanying comment.

# Editor report: Recommendation: Acceptability and feasibility of a brief intervention to enhance resilience among young people and their families in India and Kenya — R1/PR5

**DOI:** 10.1017/gmh.2024.87.pr5

**Affiliations:** School of Public Health and Preventive Medicine, Monash University Faculty of Medicine Nursing and Health Sciences, Australia

No accompanying comment.

# Editor report: Decision: Acceptability and feasibility of a brief intervention to enhance resilience among young people and their families in India and Kenya — R1/PR6

**DOI:** 10.1017/gmh.2024.87.pr6

**Affiliations:** London School of Hygiene & Tropical Medicine, United Kingdom of Great Britain and Northern Ireland

No accompanying comment.
